# Usefulness of Immature Granulocytes to Predict High Coronary SYNTAX Score in Acute Coronary Syndrome; a Cross-sectional Study

**Published:** 2020-09-15

**Authors:** Cihan Bedel, Mustafa Korkut, Fatih Aksoy, Görkem Kuş

**Affiliations:** 1 **Department of Emergency Medicine, Health Science University Antalya Training and Research Hospital, Antalya,Turkey.**; 2 **Department of Cardiology, Suleyman Demirel University Faculty of Medicine, Isparta, Turkey** **.**; 3 **Department of Cardiology, Health Science University Antalya Training and Research Hospital, Antalya,Turkey.**

**Keywords:** Inflammation, Acute coronary syndrome, Granulocytes, percutaneous coronary intervention, Emergency Medicine, Atherosclerosis

## Abstract

**Introduction:**

Immature granulocytes (IG) in peripheral blood indicate increased bone marrow activation and inflammation, and SYNTAX score (SS) is an anatomical scoring system based on coronary angiogram. This study, aimed to evaluate the relationship between IG and SS, as a new inflammatory marker in patients with acute coronary syndrome (ACS).

**Methods:**

Patients aged >18 years who were diagnosed with ACS in the emergency department were included in this study, which was planned as a cross-sectional study. Patients were divided into two groups of patients with high and low SSs according to coronary angiography results. Demographic and laboratory parameters were compared between the groups.

**Results:**

Our study consisted of 78 patients diagnosed with ACS, who met the inclusion criteria. The average age of the study group was 59 years, and 67.9% of the patients were male. 21 patients (26.9%) had high SSs and 57 patients (73.1%) had low SSs. Mean IG% was significantly higher in high SS group compared to low SS group (0.71±0.25 vs 0.44±0.21 mg/dl, p<0.001). IG% can present a high SS with 76.2% sensitivity and 75.4% specificity at a cut-off value of 0.7.

**Conclusion:**

IG was significantly higher in ACS patients with high SSs. It seems that IG can be used as a parameter, which is quickly accessible and cheap, in order to predict high SS in ACS patients in daily clinical practice.

## Introduction

Acute coronary syndrome (ACS) is one of the main reasons for admission to the emergency department and hospitalization. ACS is usually characterized by atherosclerotic plaque rupture and complete or incomplete thrombosis of the coronary arteries, which is one of the most significant causes of mortality and morbidity [1, 2]. Many pathophysiological factors influence this atherosclerotic process, and inflammation is one of these factors. Inflammation plays a significant role in initiating atherosclerosis and facilitating its progression [3]. 

Inflammatory markers, such as white blood cell (WBC), C-reactive protein (CRP), Neutrophil-lymphocyte ratio (NLR), and platelet-lymphocyte ratio (PLR), have been researched in the demonstration of poor condition in cardiovascular events [4, 5]. Immature granulocyte (IG), a parameter that is not adequately known by many clinicians, reflects the fraction of immature granulocytes in the peripheral blood. This parameter can be easily and quickly measured in automated blood cell analyzers. It has been revealed to be useful in predicting the severity of many disease processes, such as bacterial infection, acute inflammatory diseases, tissue necrosis, and acute transplant rejection, in recent years [6, 7].

Synergy between percutaneous coronary intervention with taxus and cardiac surgery (SYNTAX) score (SS) is an anatomical scoring system based on coronary angiogram, which can help with evaluating the severity of coronary artery disease and making revascularization decisions [8]. Previously, many studies have examined the relationship between WBC, CRP, Neutrophil-lymphocyte ratio (NLR), and platelet-lymphocyte ratio (PLR) and SS; however, there are no studies evaluating the relationship between IG and SS in the literature. Therefore, this study aimed to evaluate the relationship between IG and SS, as a new inflammatory marker in patients with ACS.

## Methods

***2.1. Study design and setting***

Patients aged >18, who had presented to the emergency department with pain in the chest and been admitted to the department of cardiology with a diagnosis of ACS (unstable angina pectoris/myocardial infarction without ST-segment elevation (NSTEMI)/myocardial infarction with ST-segment elevation (STEMI)), were included in this study, which was planned as a prospective cross-sectional study and performed during the period between December 01, 2019, and February 01, 2020. The diagnosis of ACS was defined as having electrocardiographic (ECG) change and/or increase of cardiac markers along with chest pain, which was assumed to be typical chest pain. In compliance with American College of Cardiology and European Society of Cardiology (ACC/ESC) criteria, STEMI was defined as ST-segment elevation in ECG and increase in all the derivations by ≥0.1mV in two consecutive derivations. Necessary approval was received from the Clinical Research Ethics Committee for the study (No: IRB-2019-355). Written informed consent was obtained from all the patients, who agreed to participate in the study. 

***Participants***

Exclusion Criteria were determined as being <18 years old, being pregnant, having a myeloproliferative disease (it may change hematological parameters), malignancy, having trauma or surgery history within the past 1 week, arrhythmia causing hemodynamic instability, heart failure, inflammatory bowel disease, granulocyte-colony stimulating factor, and using immunosuppressive agents or steroids. 

***Data gathering***

ACS patients' age, gender, history of hyperlipidemia, history of hypertension (HT), history of diabetes mellitus (DM), family history, drugs, systolic blood pressure (SBP), diastolic blood pressure (DBP), and heart rates (HR) (pulse/min) were recorded. The minimum sample size with a two-sided alpha value of 5%, a statistical power of 80% was estimated to be 50 patients. We planned to enrol a total of 80 patients, taking into account the 20% expected failure rate.

***2.2. SYNTAX Score and Angiographic Analysis***

In the study, coronary angiography (CAG) was carried out for all the patients using the Judkins technique. In order to grade the stenosis of the coronary vessels, stenoses over 50% in vessels with a size of ≥1.5mm were taken into consideration. SS was prospectively calculated by two experienced cardiologists using an algorithm based on the diagnostic angiogram. The final score was calculated using individual lesion scores by analysts who were blind to operational data and clinical outcomes. Items such as whether the stenosis was total, the level and the size of the stenosis, presence of collateral flow, presence of bifurcation or trifurcation lesion, severe folds, and severe calcification were evaluated [9, 10].

***2.3. Blood Samples***

Venous blood samples of the patients were taken within the first hour of admission to the emergency department before the primary CAG. In the samples taken during admission, WBC, neutrophil count, lymphocyte count, and IG% IG count (IGC) were measured using an automated blood analysis system (Coulter® LH 780 Hematologic Analyzer, Beckman Coulter Inc. Brea, USA). Absolute cell numbers were used in the analyses. CRP, haemoglobin, glucose values, and cardiac Troponin T levels, which were measured during the admission, were recorded. The levels of total cholesterol, high-density lipoprotein (HDL), low-density lipoprotein (LDL), and triglycerides were recorded during admission to the coronary intensive care. The left ventricular ejection fraction (LVEF) of the patients was measured using Vivid S5 (GE Healthcare, Inc. Chicago, IL, USA) device connected to 2-4 MHz transducer via Simpson's method according to the recommendations of the American Society of Echocardiography [11]. According to CAG results, patients with high SSs (>22) and patients with low SSs (≤22) were separated into two groups, and all the parameters were compared.

***2.4. Statistical Analysis***

Statistical analyses were conducted using SPSS 21.0 package program (SPSS Inc., Chicago, IL). Continuous variables were expressed as mean ± standard deviation, and categorical variables were given as number and percentage. An independent t-test was used for comparing the distribution of the parameters with normal distribution, and the Mann-Whitney U test was applied for those that did not have a normal distribution. In categorical data, the evaluation was made using the chi-square test. Logistic regression was conducted for factors associated with high SS. The optimum cut off value of IG in predicting high SS was assessed through Receiver operating characteristic (ROC) analysis. Statistical significance was defined as a p-value less than 0.05.

## Results

**Figure 1:**
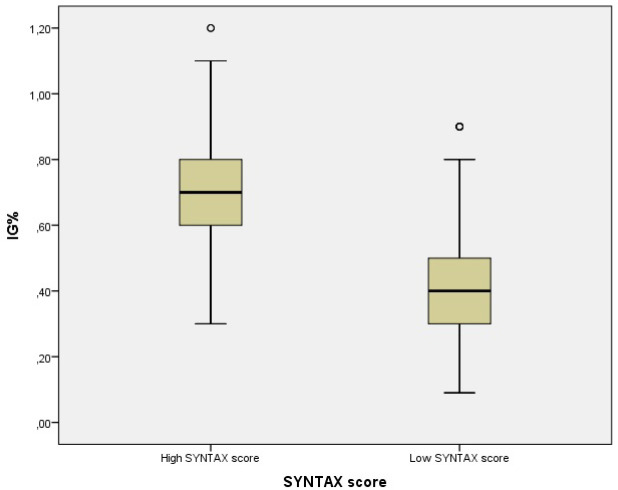
Comparison of immature granulocyte levels between low and high syntax score groups

Our study consisted of 78 patients diagnosed with ACS, who met the inclusion criteria. The average age of the study group was 59 ± 15.21 years, and 67.9% of the patients were male. 21 patients (26.9%) had high SSs (>22) and 57 patients (73.1%) had low SSs (≤22). There was no statistical difference between the groups in terms of age and gender (p>0.05). Patients with high SSs had significantly lower DBP and LVEF (p=0.006, p=0.002, respectively). Patients with high SSs had significantly higher CAD history comorbidity (p=0.009). Mean WBC, neutrophil, glucose, IGC, and CRP and troponin T levels were significantly higher in patients with high SSs. The mean IG% was significantly higher in the high SYNTAX score group compared to the low SYNTAX score group (0.71±0.25 vs 0.44±0.21mg/dl, p<0.001) (Figure 1). Besides, the percentage of mortality was significantly higher in those with high SSs (p=0.017). The demographic data and laboratory results of the groups are compared in Table 1.

**Figure 2:**
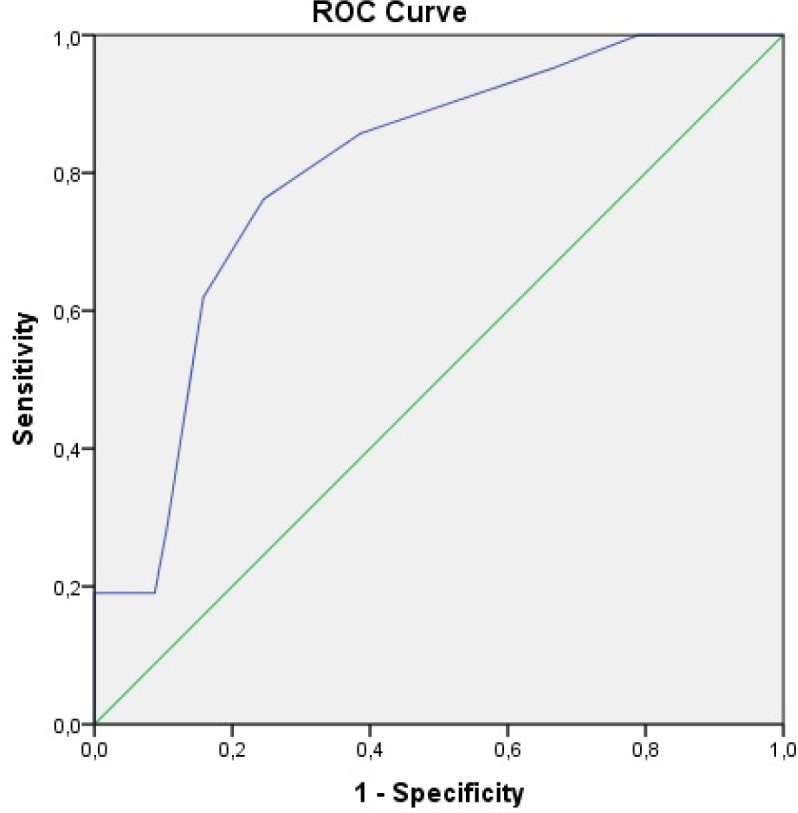
Receiver operating characteristic (ROC) curve of immature granulocyte percentage for predicting high syntax (synergy between percutaneous coronary intervention with taxus and cardiac surgery) score (p < 0.001)

22 (28.2%) patients had high IG% values (>0.6), and 56 (71.8%) patients had low IG% values (≤0.6). As seen in Table 2, the prevalence of oral antidiabetic drug use was higher at high IG% values, and mean DBP, HDL, and LDL values of patients were found to be significantly lower. In the group with high IG% levels, higher SSs were detected compared to the patients with low IG% values (17.21±14.80 vs. 10.97±8.31, p=0.024). Additionally, in the analysis of the ROC curve, IG% was shown to predict high SSs with 76.2% (95% Cl: 67.21 – 84.67) sensitivity, 75.4% (95% Cl: 66.11 – 83.81) specificity, and area under the ROC curve of 0.803 (95% CI: 0.699 - 0.908) at a cut-off value of 0.7 (Table 3, Figure 2).

## Discussion

To the best of our knowledge, this is the first study in the literature evaluating the relationship between IG and SS in ACS patients. The main findings of this study suggested that SS was independently correlated with IG%.

SS is a scoring system used to evaluate the complexity and prevalence of coronary artery disease based on CAG. It is commonly used by many physicians to specify the optimal cardiovascular treatment strategy [12, 13]. Studies have shown that patients with high SSs may have poorer cardiovascular outcomes, and the score may be an independent predictor for percutaneous interventions. Moreover, high-risk patients can be identified using this scoring system, and appropriate treatment methods can be selected [14, 15]. 

Inflammation is critically important for the initiation and progression of coronary atherosclerosis. Inflammation affects many conditions, such as endothelial dysfunction, leukocyte recruitment, and platelet activation during the atherosclerosis process [16]. Recently, it has been revealed that many inflammatory markers, such as CRP, platelet/lymphocyte ratio (PLR) and neutrophil/lymphocyte ratio (NLR), WBC, TNF-α, and cytokines can be independent risk factors for atherosclerosis. The increase in these inflammatory markers has been shown to correlate with the degree and severity of CAD [14, 17]. These inflammatory markers have been evaluated as prognostic markers for many cardiovascular diseases, such as coronary artery ectasia, stable CAD, and myocardial infarction [18, 19]. 840 patients, who underwent coronary angiography for CAD evaluation, were included in a recent study by Sahin et al. In this study, NLR was shown to be significantly associated with CAD severity in patients with STEMI, and they also reported that NLR is an independent marker for SS [2]. In a recent study by Altun et al., Troponin T and NLR were significantly associated with the angiographic severity of ACS evaluated with SS [16]. In a study conducted by Kundi et al., it was reported that the ratio of the Monocyte count to HDL could be used as a parameter that would be quickly accessible and cheap in order to predict high SS and it may be used in daily practice as well [13]. In a study conducted by Sivri et al. on 175 patients, the WBC/mean platelet volume ratio was shown to be correlated with increased SS, and thus, short- and long-term mortality [1]. IG in peripheral blood indicates increased bone marrow activation, and it can be easily measured in automated blood analyzers. It has been shown in studies that the presence of immature granulocytes in peripheral blood, which is not normally observed in healthy people, can indicate bone marrow activation and serious infection [20, 21]. Recent studies suggest that IG is correlated with prevalent intravascular coagulation and mortality in critical patients with suspected sepsis [22]. Park et al. reported that high IG values are a good diagnostic sign for severe sepsis and septic shock within the first 24 hours after admission to the intensive care unit [23]. Mathews et al. discovered that the increase in IG% was significant in appendicitis complications in the pediatric age group and only compared it with an increased CRP level and left shift [24]. In this study, we showed that patients with high IG levels had higher SSs. Besides, mortality was higher at high IG levels.

**Table 1:** Comparing the baseline characteristics of patients with high (> 22) and low (≤ 22) SYNTAX score

**Variables**	**SYNTAX** ** Score**		**P **
	**High (n=21)**	**Low ** **(n =57 )**	
**Age (years)**			
Mean ± SD	66.00 ± 16.67	57.57 ± 14.15	0.057
**Gender** n (%)			
Male	15 (68.2)	38 (66.6)	0.789
Female	6 (31.8)	19 (33.4)	
**Vital signs**			
SBP, mm Hg	131.57 ± 14.92	144.14 ± 27.01	0.071
DBP, mm Hg	83.19 ± 8.04	91.47 ± 15.26	0.006
Heart rate, beats/min	83.07 ± 15.26	84.33 ± 15.26	0.283
Ejection fraction, %	45.95 ± 12.57	55.7 ± 12.93	0.002
SYNTAX score	27.78 ± 4.79	7.38 ± 6.24	<0.001
**Previous history **			
Current smoker	12 (57.1)	29 (50.9)	0.799
Hypertension	11 (52.4)	28 (49.1)	0.500
Diabetes mellitus	7 (33.3)	22 (38.6)	0.794
Dyslipidemia	9 (42.9)	21 (36.8)	0.409
History of CAD	8 (38.1)	6 (10.5)	0.009
**Laboratory findings**			
WBC count (×10^3^/mm^3^)	13.03 ± 3.05	10.68 ± 4.15	0.003
Neutrophil, (×10^3^/mm^3^)	8.77 ± 2.84	6.84 ± 3.4	0.005
Lymphocyte, (×10^3^/mm^3^)	3.67 ± 3.20	2.97 ± 2.41	0.318
NLR	4.09 ± 3.91	3.14 ± 2.71±	0.367
PLR	112.14 ± 59.08	120.51 ± 57.22	0.499
Hemoglobin, mg/dL	13.89 ± 1.97	13.55 ± 1.97	0.355
Glucose (mg/dl	163.33 ± 71.52	140.36 ± 69.22	0.017
IGC(×10^3^/mm^3^)	0.08 ± 0.06	0.07 ± 0.01	0.004
IG%	0.71 ± 0.25	0.44 ± 0.21	<0.001
CRP (mg/dL)	35.37 ± 17.12	3.92 ± 0.54	0.021
Troponin T (ng\L)	502.00 ± 157.07	426.08 ± 157.74	0.012
**Lipid profiles (mg/dl)**			
Triglycerides	162.72 ± 92.30	212.33 ± 134.39	0.188
Total cholesterol	211.27 ± 65.80	220.64 ± 56.83	0.304
High-density lipoprotein	44.00 ± 9.01	46.28 ± 10.97	0.354
Low-density lipoprotein	135.50 ± 57.34	134.66 ± 45.95	0.650
**Previous medication** n (%)			
RAS blocker	2 (9.5)	5 (8.8)	0.611
ACE-I	2 (9.5)	14 (24.6)	0.210
Beta blocker	3 (14.3)	15 (26.3)	0.368
Diuretic	4 (19)	9 (15.8)	0.740
Calcium channel blocker	6 (28.6)	8 (14)	0.184
Statin	10 (47.6)	22 (38.6)	0.605
Antiaggregant	5 (23.8)	12 (21.1)	0.766
Oral antidiabetic drug	4 (19)	16 (28.1)	0.562
** Mortality**			
Number (%)	3 (14.3)	0 (0)	0.017

Data are presented as mean ± standard deviation or frequency (%). SYNTAX score: Synergy between percutaneous coronary intervention with taxus and cardiac surgery; SBP: Systolic Blood Pressure; DBP: Diastolic Blood Pressure; CAD: Coronary artery disease; WBC: white blood cell; NLR: neutrophil lymphocyte ratio; PLR: platelet lymphocyte ratio; IGC: Immature granulocyte count; IG%: Immature granulocyte percentage; CRP: C-reactive protein; RAS: Renin–angiotensin system; ACE-I: Angiotensin converting enzyme inhibitor.

**Table 2:** Comparing the baseline characteristics of patients with high (> 0.6) and low (≤ 0.6) immature granulocyte

**Variables**	**Immature granulocyte**		**P **
	**High (n=22)**	**Low (n =56 )**	
**Age (years)**			
Mean ± SD	61.13 ± 20.28	59.33 ± 12.92	0.807
**Gender n (%)**			
Male	15 (68.2)	38 (66.6)	0.601
Female	6 (31.8)	19 (33.4)	
**Vital signs**			
SBP, mm Hg	129.45 ± 16.31	145.19 ± 26.37	0.07
DBP, mm Hg	83.54 ± 9.18	91.48 ± 15.15	0.024
Heart rate, beats/min	84.81 ± 18.21	82.85 ± 8.92	0.367
Ejection fraction, %	50.90 ± 11.51	54.01 ± 10.51	0.234
SYNTAX score	17.21 ± 14.80	10.97 ± 8.31	0.024
**Previous history **			
Current smoker	13 (59.1)	28 (50)	0.615
Hypertension	10 (45.5)	29 (51.8)	0.802
Diabetes mellitus	7 (31.8)	22 (39.3)	0.610
Dyslipidemia	5 (22.7)	25 (44.6)	0.120
History of CAD	5 (22.7)	9 (16.1)	0.522
**Laboratory findings**			
WBC count (×10^3^/mm^3^)	12.88 ± 3.92	10.70 ± 3.90	0.032
Neutrophil, (×10^3^/mm^3^)	9.26 ± 3.78	6.61 ± 2.87	0.003
Lymphocyte, (×10^3^/mm^3^)	3.70 ± 0.82	2.97 ± 0.96	0.526
NLR	4.22 ± 4.11	3.06 ± 2.73	0.111
PLR	112.78 ± 61.46	119.95 ± 56.66	0.560
Hemoglobin, mg/dL	12.95 ± 2.25	13.89 ± 1.78	0.131
Glucose (mg/dl	114.38 ± 63.50	148.07 ± 73.39	0.556
CRP (mg/dL)	24.71 ± 14.98	7.35 ± 3.01	0.693
Troponin T (ng\L)	519.66 ± 156.17	424.96 ± 160.55	0.011
**Lipid profiles (mg/dl)**			
Triglycerides	175.11 ± 107.80	208.35 ± 132.05	0.381
Total cholesterol	189.22 ± 41.98	227.73 ± 60.65	0.012
High-density lipoprotein	42.38 ± 8.90	46.80 ± 10.84	0.011
Low-density lipoprotein	118.77 ± 31.18	140.03 ± 52.11	0.08
**Previous medication n (%)**			
RAS blocker	2 (9.1)	5 (8.9)	0.561
ACE-I	3 (13.6)	13 (23.2)	0.535
Beta blocker	4 (18.2)	14 (25)	0.766
Diuretic	4 (18.2)	9 (16.1)	1.000
Calcium channel blocker	5 (22.7)	9 (16.1)	0.522
Statin	6 (27.3)	26 (46.4)	0.135
Antiaggregant	5 (22.7)	12 (21.4)	1.000
Oral antidiabetic drug	2 (9.1)	18 (32.1)	0.45
** Mortality**			
Number (%)	3 (13.6)	0 (0)	0.02

Data are presented as mean ± standard deviation (SD) or frequency (%). SBP: Systolic Blood Pressure; DBP: Diastolic Blood Pressure; SYNTAX score: Synergy between percutaneous coronary intervention with taxus and cardiac surgery; CAD: Coronary artery disease; WBC: white blood cell; NLR: neutrophil lymphocyte ratio; PLR: platelet lymphocyte ratio; IGC: Immature granulocyte count; IG%: Immature granulocyte percentage; CRP: C-reactive protein; RAS: Renin–angiotensin system; ACE-I: Angiotensin converting enzyme inhibitor.

**Table 3:** Screening performance characteristics of immature granulocyte percentage in predicting the SYNTAX (synergy between percutaneous coronary intervention with taxus and cardiac surgery) score in 0.7 cut off point

**Characters**	**Value (95% CI)**	**Characters**	**Value (95% CI)**
**Sensitivity**	76.2 (67.21-84.67)	**NPV**	76.5 (69.15 – 82.59
**Specificity**	75.4 (66.11-83.81)	**PLR**	3.17 ( 2.2 - 4.57)
**PPV**	76 (68.74 - 82.02)	**NLR**	0.31 (0.21 –0.45)

Confidence interval (CI). PPV: Positive predictive value; NPV: Negative predictive value; PLR: Positive likelihood ratio; NLR: Negative likelihood ratio.

## Limitations

The first limitation was that our study, although it was designed prospectively, was conducted with a small number of patients due to the COVID-19 pandemic. Furthermore, the mono-center design of our study increases bias. As another limitation, the fact that the decision on CAG was not made by the same physicians may have influenced the results. Additionally, the period from the emergence of the symptoms until hospital admission could not be assessed, which may affect the values of inflammatory markers. Finally, since patients with a history of CABG were not included in the study, SS could not be confirmed in this population.

## Conclusion

IG was significantly higher in ACS patients with high Syntax scores. It seems that IG can be used as a parameter, which is quickly accessible and cheap, in order to predict the high SYNTAX score in ACS patients in daily clinical practice.

## References

[1] Sivri S (2019). Usefulness of white blood cell count to mean platelet volume ratio in the prediction of SYNTAX score in patients with non-ST elevation myocardial infarction. Pakistan journal of medical sciences,.

[2] Şahin DY (2013). Neutrophil to lymphocyte ratio is associated with the severity of coronary artery disease in patients with ST-segment elevation myocardial infarction. Angiology.

[3] Moriya J (2019). Critical roles of inflammation in atherosclerosis. Journal of cardiology.

[4] Sari I (2015). Relation of neutrophil-to-lymphocyte and platelet-to-lymphocyte ratio with coronary artery disease severity in patients undergoing coronary angiography. Kardiologia Polska (Polish Heart Journal).

[5] Silvestre-Roig C (2020). Neutrophils as regulators of cardiovascular inflammation. Nature Reviews Cardiology.

[6] Karon BS (2017). Evaluation of lactate, white blood cell count, neutrophil count, procalcitonin and immature granulocyte count as biomarkers for sepsis in emergency department patients. Clinical biochemistry.

[7] Lima L R (2019). Automated immature granulocyte count in patients of the intensive care unit with suspected infection. Jornal Brasileiro de Patologia e Medicina Laboratorial.

[8] Collet C (2017). Integration of non-invasive functional assessments with anatomical risk stratification in complex coronary artery disease: the non-invasive functional SYNTAX score. Cardiovascular diagnosis and therapy.

[9] Sianos G (2005). The SYNTAX Score: an angiographic tool grading the complexity of coronary artery disease. EuroIntervention.

[10] Neumann F (2019). 2018 ESC/EACTS guidelines on myocardial revascularization. European heart journal.

[11] Schiller N B (1989). Recommendations for quantitation of the left ventricle by two-dimensional echocardiography. Journal of the American Society of Echocardiography,.

[12] Cerit L, Z Cerit (2019). Vitamin D Deficiency is not Associated with Higher Levels of SYNTAX Score. Brazilian journal of cardiovascular surgery,.

[13] Kundi, H (2016). Association of monocyte/HDL-C ratio with SYNTAX scores in patients with stable coronary artery disease. Herz.

[14] Kurtul S ( 2014). Neutrophil to lymphocyte ratio predicts SYNTAX score in patients with non-ST segment elevation myocardial infarction. International Heart Journal.

[15] Zuin M (2017). Correlation and prognostic role of neutrophil to lymphocyte ratio and SYNTAX score in patients with acute myocardial infarction treated with percutaneous coronary intervention: a six-year experience. Cardiovascular Revascularization Medicine,.

[16] Altun B (2014). The relationship between high-sensitive troponin T, neutrophil lymphocyte ratio and SYNTAX Score. Scandinavian Journal of Clinical and Laboratory Investigation,.

[17] Kurtul A (2014). Association of platelet-to-lymphocyte ratio with severity and complexity of coronary artery disease in patients with acute coronary syndromes. The American journal of cardiology,.

[18] Sarli B (2014). Neutrophil-to-lymphocyte ratio is associated with severity of coronary artery ectasia. Angiology.

[19] Azab B (2010). Usefulness of neutrophil to lymphocyte ratio in predicting short-and long-term mortality after non–ST-elevation myocardial infarction. The American journal of cardiology.

[20] Park J H (2017). Delta neutrophil index (DNI) as a novel diagnostic and prognostic marker of infection: a systematic review and meta-analysis. Inflammation Research,.

[21] Senthilnayagam B (2012). Automated measurement of immature granulocytes: performance characteristics and utility in routine clinical practice. Pathology research international,.

[22] Seok Y (2012). Delta neutrophil index: a promising diagnostic and prognostic marker for sepsis. Shock.

[23] Park B H (2011). Delta neutrophil index as an early marker of disease severity in critically ill patients with sepsis. BMC infectious diseases.

[24] Mathews, E K (2014). Utility of immature granulocyte percentage in pediatric appendicitis. journal of surgical research.

